# Supplementary material for: Preferences and constraints: the value of economic games for studying human behaviour
Source: R Soc Open Sci. 2020 Jun 3;7(6):192090. doi: 10.1098/rsos.192090 (PMC7353969; doi:10.1098/rsos.192090)
Supplement: Supplementary material [file rsos192090supp1.pdf]

Supplementary Materials to: "Preferences and constraints: The value of economic games for studying human behavior"

Anne C. Pisor[1,2], Matthew M. Gervais[3,4], Benjamin G. Purzycki[2,5], & Cody T. Ross[2]

[1]*Department of Anthropology. Washington State University. Pullman, USA.*
[2]*Department of Human Behavior, Ecology, and Culture. Max Planck Institute for Evolutionary Anthropology. Leipzig, Germany.*
[3]*Department of Psychology. University of British Columbia. Vancouver, Canada.*
[4]*Department of Psychology. Simon Fraser University. Burnaby, Canada.*
[5]*Department of the Study of Religion. Aarhus University. Aarhus, Denmark.*

Contents

1 Study site . . . 2
2 Data . . . 2
3 Modeling . . . 5
4 Results . . . 7
5 Discussion . . . 19

1 Study site

This study was conducted in a coastal site ($n = 116$) in Colombia. The population is composed of a majority of Afrocolombians, along with minorities of Mestizos and indigenous Emberá. Like many other communities in the region, this community has been heavily affected by Colombia's internal conflicts. A large proportion of residents in the site are considered internally displaced persons within Colombia, having resettled after being forced from their natal communities. This is important because it provides a social context in which the establishment and maintenance of social relationships is critical in buffering the resource shocks associated with high levels of poverty and forced displacement. In terms of subsistence, the community currently relies on a mixture of fishing and local wage labor. However, hunting, horticulture, and animal husbandry are also practiced.

Informed consent was obtained from each respondent and the community leader (when appropriate) prior to data collection. Because of limited literacy rates at the study site, informed consent was obtained verbally. All field protocols were approved by the Max Planck Institute for Evolutionary Anthropology, Department of Human Behavior, Ecology and Culture, and declared exempt from additional IRB oversight.

2 Data

For all demographic and survey response data, individuals were interviewed in the winter of 2016. Economic games were played in the winter of 2017.

Outcome data

Economic game data We used three network-structured economic games[1]: an allocation game, a taking game, and costly punishment game. For each of these games, we presented individuals with a photo array containing 7x10 cm photographs of all interviewed male and female adults residing in the field-site during the winter of 2016. In total there were 116 alters (recipients) to whom focal players (deciders) could allocate coins or tokens. These photos were organized onto four 35x50 cm boards. The positions of the boards were randomized between respondents, and the order of the photographs on the boards was randomized on four separate occasions over the course of data collection. In total, 93 respondents completed the economic games. All three games were played in sequence—in the same order (allocation, taking, and costly punishment)—during the same interview.

After all interviews were complete, all game participants were given the currency allocated to them by themselves and other community members during the games. Individuals who appeared as alters but who could not be found to participate as focal players (normally due to out-migration from the community) were not allocated payouts—instead, transfers directed to these players were refunded to the focal players who made such transfers. Total stakes per person amounted to 82,500 Colombian pesos ($\sim$27 USD) at the time of data collection. Using self reported income at the household-level over the month prior to the initial 2016 survey—and assuming that 21 (five of every seven) of these days were work days—mean daily household income is 82,700 Colombian pesos. However, there is significant inequality in income, as the median daily household income is only 50,900 Colombian pesos.

In the allocation game, the stakes were set at fifteen 1,000 peso coins (15,000 pesos total). Individuals could allocate any number of these fifteen coins to any cell in the photo array, including their own. Individuals varied widely in

how much was kept and how much was given, with a mean giving rate of 11,760 (78.4%), a median of 13,000 (86.6%), a standard deviation of 3,500, a minimum of 0, and a maximum of 15,000 pesos.

In the taking game, an initial allocation of one 500 peso coin to each photo was provided by the researcher for a total stakes of 57,500 pesos; participants could leave the 500 peso coin placed by the researcher on each photo or take it for themselves. Again, individuals varied widely in how much was taken and how much was left, with a mean leaving rate of 39,800 (69.2%) pesos, a median of 47,000 (81.7%), a standard deviation of 17,600, a minimum of 0, and a maximum of 57,500 pesos.

In the costly punishment game, the stakes were set at 10,000 pesos (ten 1,000 peso coins), which were allocated to the recipient. Individuals could keep the coins or use them purchase red tokens to punish/reduce other community members. Each token cost 1,000 pesos, and led to a reduction of the alter's income by 4,000 pesos—the same multiplier used elsewhere[1]. Punishment was fairly infrequent, with a mean payment rate for punishing of 1,600 (16%), a median of 0, a standard deviation of 2,800, a minimum of 0, and a maximum of 10,000 pesos.

Food or money transfers Transfer ties between each pair of individuals were assessed by asking each individual to name all individuals to whom they have given food or money in the last 30 days, and all individuals who have given them food or money over the same time period. This question was asked as part of the social network battery conducted in the winter of 2016.

Covariates

We consider fifteen variables that might play a role in explaining variation in economic game play and resource transfers in our statistical models. In order to normalize the effects of our shrinkage priors, we divide each of these variables by their respective maximums before model fitting. Missing data were handled using the "mean imputation" technique: missing data were imputed a single time prior to model fitting using the mean or median of the distribution for the relevant variable.

1) *Friendship*

Friendship ties between each pair of individuals were assessed by asking each individual to name all individuals with whom they have spent time socializing in the last 30 days. This question was asked as part of the social network battery conducted in the winter of 2016.

2) *Marriage*

Marriage ties between each pair of individuals were assessed by asking each individual to name all individuals with whom they are currently married. This question was asked as part of the marriage history survey conducted in the winter of 2016.

3) *Relatedness*

Relatedness ties between each pair of individuals were created by first asking each individual in the community to name all parents and children. A community-wide pedigree was then constructed and used to create a pairwise matrix of relatedness values. These data were collected as part of the reproductive history survey conducted in the winter of 2016.

4) *Age*

Age is typically based on self-reported date of birth. In the majority of cases, individuals know their date of birth from their national ID, or presented their ID card to the research team.

In a small set of cases, especially among the elderly and indigenous sub-samples, age is only a self-reported estimate. Data were collected in winter of 2016.

5) *Ethnicity as indigenous*

A binary indicator for identity as Emberá Chamí or a related group. Data were collected in winter of 2016.

6) *Sex*

A binary indicator for identity as male. Data were collected in winter of 2016.

7) *Out-migration*

A binary indicator for individuals who were present in the community in winter 2016, but who were not present in the community in winter 2017 during the economic games (and could not be found to play the game, despite appearing as alters). Many of these individuals were reported to have been involved in activities damaging to the local community by the residents who remained.

8) *Depression*

A Spanish language implementation of the Kessler Psychological Distress Scale[2] (K6) was presented to each respondent in the study site in winter 2016. An individual was classified as depressed if they responded that they were *often* or *always* depressed over the preceding 30 days. This was treated as a binary 0 or 1 outcome.

9) *Same ethnicity*

A binary indicator if individuals i and j (that is, the decider and the recipient) are either both indigenous or both non-indigenous. If both respondents were of the same ethnicity, this value is 1; if one respondent was non-indigenous and the other indigenous, this value is 0.

10) *Same sex*

A binary indicator if individuals i and j are either both male or both female. If both respondents were of the same sex, this value is 1; if one respondent was male and the other female, this value is 0.

11) *Material wealth*

As our primary measure of economic stability, we use data on the household wealth of each focal individual in winter of 2016. This variable is composed of the sum total of the local monetary value of all: cars, trucks, motorcycles, mototaxis, motorboats, canoes, computers, TVs, washing machines, refrigerator, stoves, microwaves, cell phones, cows, pigs, and chickens in the household of the focal respondent.

12) *Unable to work*

Some individuals are unable to work to provide for themselves and their families. Ability to work is a binary measure based on a qualitative assessment by CTR. Those individuals with limited ability to work include some, but not all, elderly residents, as well as those individuals who have suffered injuries that prevent them from working. Data were coded in winter of 2017.

13) *Food insecurity*

Food insecurity was assessed with the question: how many days in the last month did you have so little food that you or someone in your family had go to bed hungry? Respondents indicating that someone in their household went to bed hungry for one or more days were coded as food insecure. This is a binary variable collected in winter of 2016.

14) *Grip strength*

Grip strength was assessed using a Camry

Digital Hand Dynamometer. Two readings were taken on each hand, and the average of all four ratings was used as our measure of grip strength. Data were collected in winter of 2016.

15) *Reciprocation*

In our most basic model, we use a simplified version of the social relations model (SRM),[3] but omit dyadic random effects and include the transpose of the outcome matrix as a dyadic predictor. This captures reciprocity of giving in the self-reported transfers and RICH allocation game, reciprocity of leaving in the RICH taking game, and reciprocity of punishment in the RICH costly reduction game. We also do a robustness check that instead uses the full SRM with dyadic random effects in order to avoid using the transposed outcome matrices, as the simpler models can suffer from residual confounding.

3 Modeling

Let $A_{[i,1:J]} \in \mathbb{N}^J$ be a vector of coin allocations or transfer ties by individual i across J alters. We can model these outcomes using a multinomial regression model:

$$A_{[i,1:J]} \sim \text{Multinomial}(\text{Softmax}(\theta_{[i,1:J]})) \quad (1)$$

where the Softmax function maps $\theta_{[i,1:J]} \in \mathbb{R}^J$ to a unit J-simplex, which gives the probability of an allocation to each alter. To parameterize the model, we first define intermediate variables. The effects of covariates linked to a focal individual are defined as:

$$\psi_{[i]} = \lambda_{[i]} + \alpha_{[0]} + \alpha_{[1]}X_{[i]} + \alpha_{[2]}Y_{[i]} + \dots \quad (2)$$

The effects of covariates linked to alters are defined as:

$$\phi_{[i,1:J]} = \pi_{[1:J]} + \beta_{[1]}X_{[1:J]} + \beta_{[2]}Y_{[1:J]} \dots \quad (3)$$

And, the effects of covariates linked to dyads are defined as:

$$\kappa_{[i,1:J]} = \delta_{[i,1:J]} + \gamma_{[1]}Z_{[i,1:J]} + \dots \quad (4)$$

We can then define $\theta_{[i,1:J]}$ as:

$$\theta_{[i,1:J]} = \big(\psi_{[i]} + \big(\phi_{[i,1:J]} + \kappa_{[i,1:J]}\big)\big) \circ Q_{[i,1:J]} \quad (5)$$

Here X and Y are covariate vectors, while Z is a matrix. This implies that $\psi_{[i]}$ is a scalar, and that $\phi_{[i]}$ and $\kappa_{[i]}$ are J-vectors. Finally, Q is a $J \times J$ matrix with ones on the off-diagonals and zeros on the diagonal, and serves as an indicator for *focal* and *alter* cases; in other words, Q indicates which individual is focal and which individuals are alters in each row. The symbol $\circ$ denotes the Hadamard product, which leads to the ith cell in $\theta_{[i]}$ being set to zero. As such, the coefficients on the predictor variables represent the change in log-odds of an allocation to an alter, relative to an allocation to self. The parameters λ and π are both J-vectors and serve as random effects for focal and alter, respectively. The parameter matrix δ is a dyad-level random effect.

In the allocation game model, $A_{[i,1:J]}$ represents the number of coins placed by focal individual i on the photographs of alters $1, \dots, J$, where the photograph of individual i is included in the set of J photographs (individuals can allocate to themselves by placing coins on their own photos). In the taking game model, $A_{[i,1:J]}$ represents the number of coins left by individual i on the photographs of alters $1, \dots, J$—this is limited by the study design to be either a single coin or nothing, with the exception of the photograph of the focal individual ($A_{[i,i]}$), who will have the sum total of coins taken from alters. In the costly punishment model, $A_{[i,1:J]}$ represents the number of punishment tokens placed by focal individual i on the photographs of alters $1, \dots, J$—with the exception that $A_{[i,i]}$ represents the number of coins kept by individual i and not allocated to punishment.

Finally, in the food and money transfer model, $A_{[i,1:J]}$ represents the directed ties between individual i and alters $1,\ldots,J$—this is limited by the study design to be a binary indicator of a tie existing. In this outcome, $A_{[i,i]}$ is set as: $J - \sum_{j \neq i} A_{[i,j]}$; i.e., the number of ties not made to alters in the community. This reflects the empirical fact that the total number of possible ties in the outcome vector is constant across individuals in the community, and keeps the data structure of the outcomes consistent across games.

Truncated Multinomial Robustness check Note that the leaving and food/money transfer outcomes are not true multinomial allocations, since there is a maximum value of 1 on the off-diagonals. To properly account for this game constraint, we consider a robustness check in which Eq. 1 is replaced by the appropriate truncated multinomial distribution. First, we define:

$$\Theta_{[1:J]} = \text{Softmax}(\theta_{[i,1:J]}) \quad (6)$$

and, like in Eq. 1, we model the outcome vectors as:

$$A_{[i,1:J]} \sim \text{Multinomial}(\Theta_{[1:J]}) \quad (7)$$

To account for the constrained nature of the taking game and self-reported transfer outcomes, we then renormalize the probability mass function by dividing by the cumulative probability of legal allocations given the allocation constraints. For notational convenience, assume that the self is category J in the following text, and let $x_{[k,1:J]}$ be a row from the matrix containing all 2^{J-1} legal move combinations—e.g., $x_{[1,1:J]} = (0,0,\ldots,J)$, $x_{[2,1:J]} = (1,0,\ldots,J-1)$, and $x_{[2^{J-1},1:J]} = (1,1,\ldots,1)$. Note that $\sum_{j=1}^{J} x_{[k,j]} = J$ for all k. Then, Ψ gives the cumulative probability mass of legal moves:

$$\Psi = \sum_{k=1}^{2^{J-1}} \frac{J!}{x_{[k,1]}!x_{[k,2]}!\ldots x_{[k,J]}!} \Theta_{[1]}^{x_{[k,1]}} \Theta_{[2]}^{x_{[k,2]}} \ldots \Theta_{[J]}^{x_{[k,J]}} \quad (8)$$

Because Stan only needs the log probability up to a proportion, we can omit the constant terms, and calculate just Ψ^*:

$$\begin{aligned}\Psi^* &= \sum_{k=1}^{2^{J-1}} \Theta_{[1]}^{x_{[k,1]}} \Theta_{[2]}^{x_{[k,2]}} \ldots \Theta_{[J]}^{x_{[k,J]}} \quad (9)\\ &= (\Theta_{[1]} + \Theta_{[J]}) \ldots (\Theta_{[J-1]} + \Theta_{[J]})(0 + \Theta_{[J]})\\ &= \Theta_{[J]} \prod_{j=1}^{J-1} (\Theta_{[j]} + \Theta_{[J]})\end{aligned}$$

Taking logs we get:

$$\log(\Psi^*) = \log(\Theta_{[J]}) + \sum_{j=1}^{J-1} \log(\Theta_{[j]} + \Theta_{[J]}) \quad (10)$$

The target in Stan is then decremented by $\log(\Psi^*)$ after each call of Eq. 7.

Priors Because both models are heavily parameterized relative to the number of individuals in the sample, we use regularizing priors on all top-level parameters:

$$\alpha \sim \text{Normal}(0, 1.5) \quad (11)$$

$$\beta \sim \text{Normal}(0, 1.5) \quad (12)$$

$$\gamma \sim \text{Normal}(0, 1.5) \quad (13)$$

These priors shrink effects towards zero, reducing effective parameter complexity.

Simplified SRM

In the simplified version of the SRM, where we include the transpose of the outcome matrix as a dyadic predictor, wet set $\delta_{[i,j]} = 0$ for all i and j, and then give the other random effects standard priors:

$$\lambda \sim \text{Normal}(0, \sigma_\lambda) \tag{14}$$

$$\pi \sim \text{Normal}(0, \sigma_\pi) \tag{15}$$

where:

$$\sigma_\lambda \sim \text{Exponential}(1.5) \tag{16}$$

$$\sigma_\pi \sim \text{Exponential}(1.5) \tag{17}$$

Full SRM

In the full social relations model, we instead use the more complex pooling strucuture outlined in McElreath's *Statistical Rethinking*[4]:

$$\begin{pmatrix} \lambda_{[i]} \\ \pi_{[i]} \end{pmatrix} \sim \text{MV Normal}\left(\begin{pmatrix} 0 \\ 0 \end{pmatrix}, \begin{pmatrix} \sigma_\lambda^2 & \sigma_\pi\sigma_\lambda\rho \\ \sigma_\lambda\sigma_\pi\rho & \sigma_\pi^2 \end{pmatrix} \right) \tag{18}$$

which, computationally, is better to implement by defining:

$$\begin{pmatrix} \lambda_{[i]} \\ \pi_{[i]} \end{pmatrix} = \begin{pmatrix} \sigma_\lambda \\ \sigma_\pi \end{pmatrix} \circ \left(L * \begin{pmatrix} \hat{\lambda}_{[i]} \\ \hat{\pi}_{[i]} \end{pmatrix} \right) \tag{19}$$

$$\sigma_\lambda \sim \text{Exponential}(1.5) \tag{20}$$

$$\sigma_\pi \sim \text{Exponential}(1.5) \tag{21}$$

$$\hat{\lambda}_{[i]} \sim \text{Normal}(0, 1) \tag{22}$$

$$\hat{\pi}_{[i]} \sim \text{Normal}(0, 1) \tag{23}$$

$$L \sim \text{LKJ Cholesky}(2) \tag{24}$$

where L is a Cholesky factor from the decomposition of the 2×2 correlation matrix with ρ on the off-diagonal.

We use this same approach for the dyad-level random effects:

$$\begin{pmatrix} \delta_{[i,j]} \\ \delta_{[j,i]} \end{pmatrix} = \begin{pmatrix} \sigma_\delta \\ \sigma_\delta \end{pmatrix} \circ \left(L_\delta * \begin{pmatrix} \hat{\delta}_{[i,j]} \\ \hat{\delta}_{[j,i]} \end{pmatrix} \right) \tag{25}$$

$$\sigma_\delta \sim \text{Exponential}(1.5) \tag{26}$$

$$\hat{\delta}_{[i,j]} \sim \text{Normal}(0, 1) \tag{27}$$

$$L_\delta \sim \text{LKJ Cholesky}(2) \tag{28}$$

Under this model, ρ gives a measure of generalized reciprocity—those who give more (to anyone) also receive more (from anyone)—and ρ_δ gives a measure of dyadic reciprocity—if focal i gives to alter j, then j is also more likely to give to i.

Software Data analysis was handled entirely in R (version 3.6.0)[5]. Statistical models were coded in Stan and fit using the RStan package (Version 2.18.2)[6]. We diagnosed model fits and Markov Chain Monte Carlo performance using trace plots, $\hat{R}$, and reported effective samples[7]. See Figures 1 and 2 for a sample of trace plots from each model. All diagnostics indicated good model fit for both standard and truncated multinomial models. Note, however, that in the case of the taking/leaving game under the full SRM with truncated multinomial outcome distribution, we could not get a good MCMC fit; as such, we do not present that model. The geometry of the posterior distribution became too difficult for Stan to sample from, yielding divergent MCMC transitions. Code and data for diagnostics and analysis replication are provided in the Supplementary Materials and will be maintained on GitHub at: www.github.com/ctross/preferencesandconstraints

4 Results

Here we present the results of an analysis of the three RICH games alongside the results of a similar analysis of self-reported resource transfers.

Simplified SRM Figures 3 and 4 show the standardized (scaled such that the posterior confi-

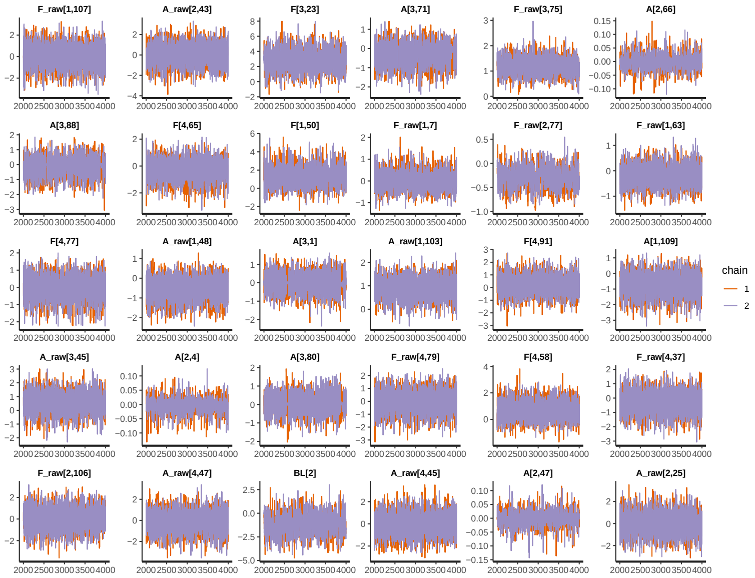

(a) Standard multinomial model, simplified SRM

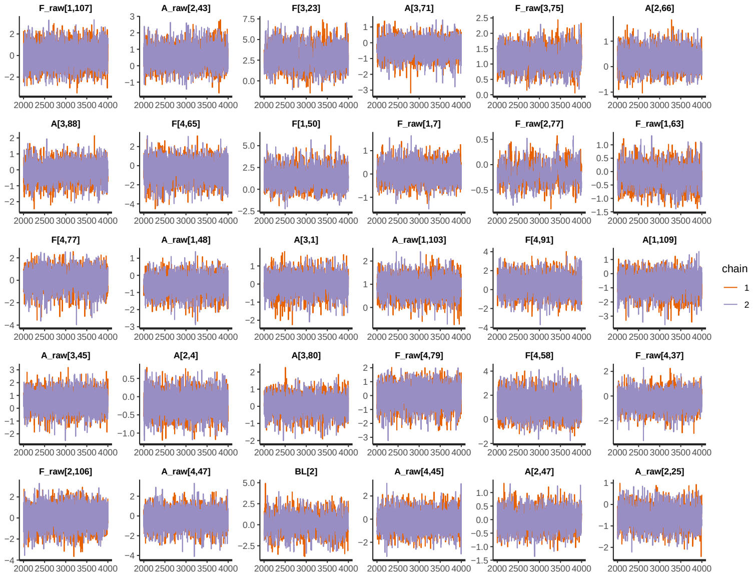

(b) Truncated multinomial model, simplified SRM

Figure 1: Traceplots for a random sample of posterior parameter estimates from the simplified social relations model. These illustrate good mixing, and convergence of two chains to similar posterior regions.

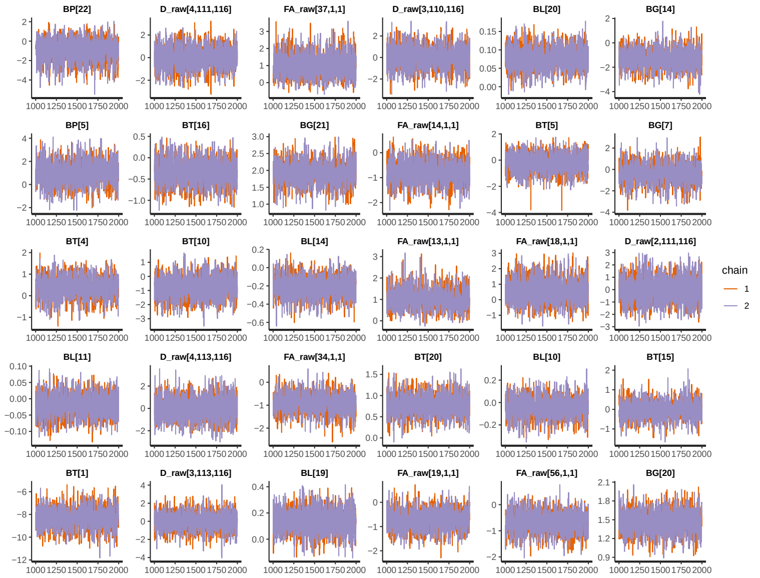

(a) Standard multinomial model, full SRM

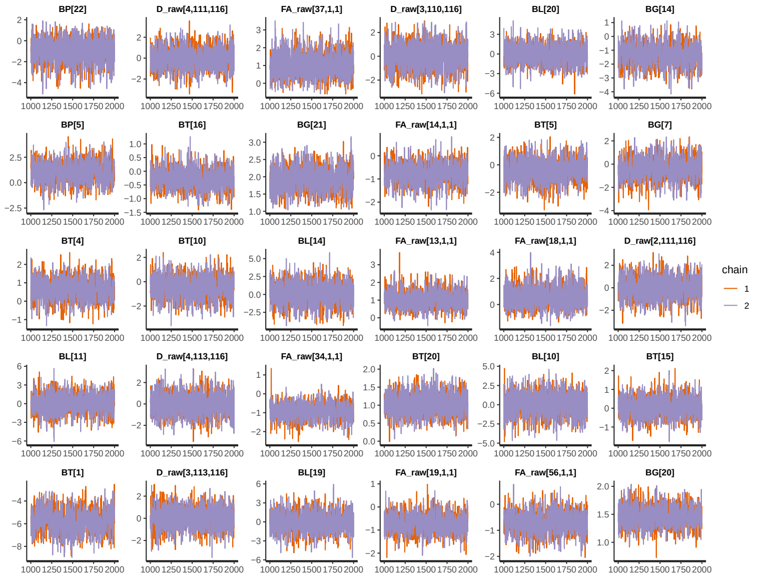

(b) Truncated multinomial model, full SRM

Figure 2: Traceplots for a random sample of posterior parameter estimates from the full social relations model. These illustrate good mixing, and convergence of two chains to similar posterior regions.

dence regions are of equal width) and raw estimates, respectively, from the standard multinomial model. Figures 5 and 6 show the standardized and raw estimates, respectively, from the truncated multinomial model. In each figure, each column represents a single outcome variable/statistical model, and each row represents a predictor variable of that outcome. Rows are broken into blocks illustrating the effects of decider/focal characteristics, alter characteristics, and dyadic characteristics. The results are very similar between the standard and truncated models.

Full SRM Figures 7 and 8 show the standardized and raw estimates, respectively, from the standard multinomial model using the full SRM. Figures 9 and 10 show the standardized and raw estimates, respectively, from the truncated multinomial model using the full SRM. As before, in each figure, each column represents a single outcome variable/statistical model, and each row represents a predictor variable of that outcome. Rows are broken into blocks illustrating the effects of decider/focal characteristics, alter characteristics, and dyadic characteristics. The results are very similar between the standard and truncated models, and between the full and simplified SRM, so we reference Figure 7 in the text.

Main findings

There are two key points to note. First, classic dyadic factors such as kinship, friendship, coethnicity, and to a lesser degree, reciprocity are associated positively with both self-reported resource transfers and experimental allocations. Likewise, some focal (e.g., age) and alter characteristics (e.g., out-migration) are also consistent between the models of self-reported resource transfers and experimental allocations, demonstrating that behavior in the allocation and taking games parallels—at least to some extent—behavior in a corresponding "real world" context.

Second, there are some notable differences between predictors of experimental transfers and self-reported resource transfers, possibly demonstrating that these experimental games allow respondents more freedom to act on preferences than they have in "real world" contexts. For example, food insecure focals are less likely to report giving food or money to others, but are no less likely than the average person to give or leave coins for others in the experimental games, possibly reflecting a preference to reciprocate in kind to alters with whom they have not been able to, due to low resource availability. Similarly, individuals who cannot work do not report receiving food or money transfers more than other individuals but are preferential targets of experimental giving. Jointly, these results both support the ecological validity of RICH games and demonstrate that experimental games can measure preferences in a way that self-report and observational studies sometimes cannot.

Detailed explanation of all findings

All text below refers to Figure 7.

Transfers In the first column, top block, we see that food insecure, elderly, and depressed individuals are significantly less likely to report transferring food or money to other community members. In the next block (first column second row), we see that alters who out-migrated from the community between 2016 and 2017 were less likely to be given allocations, as were those with high grip strength. Note also that the resource transfer question was asked in 2016, prior to any out-migrations, so the negative effect estimated here likely reflects the dissolving of social bonds that preceded excommunication or out-migration. Finally, we see in the third block that a resource transfer is more likely between individuals of the same ethnicity, as well

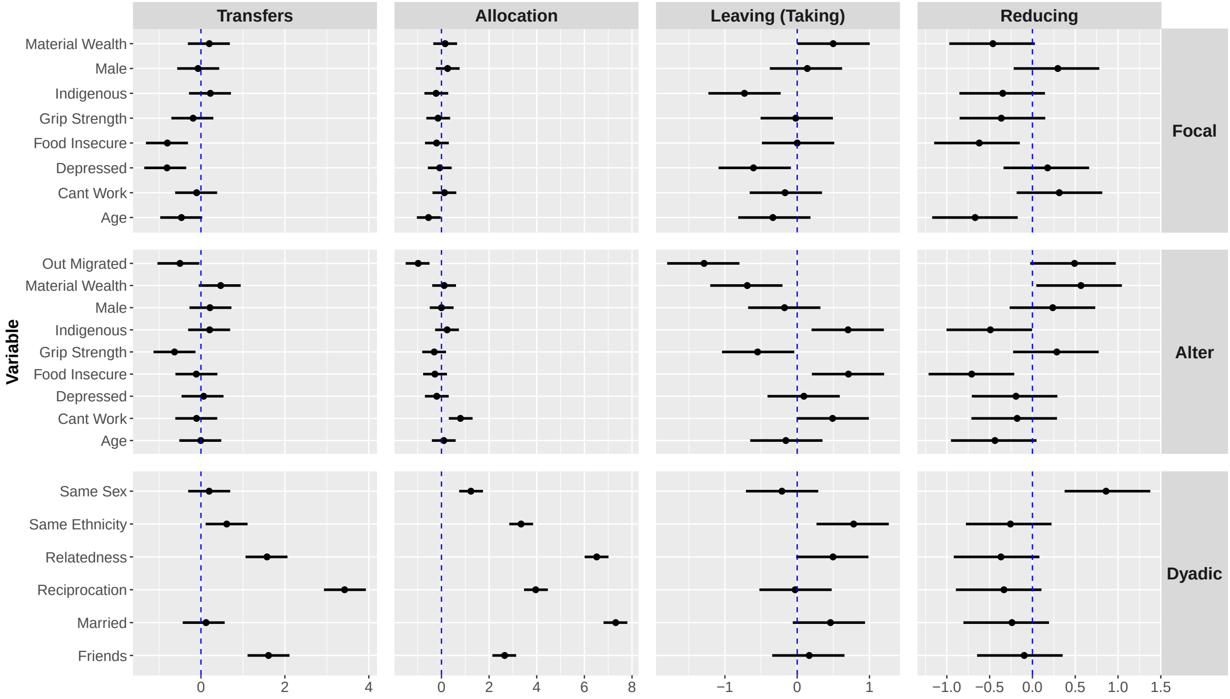

Figure 3: Multinomial regression results (standardized coefficients) from the simplified SRM. Multivariate model estimates (medians and 90% credible regions) of predictors of allocations to alters. Each column indicates an outcome variable: from left to right, i) resource transfers over the 30 days prior to the survey, ii) coin allocations in the allocation game, iii) coin allocations in the taking game (coded so that positive parameter estimates reflect *leaving* coins), and iv) token allocations in the reducing game. The top block of estimates in each model gives the effects of focal characteristics on the probability of allocating to alters. The second block of estimates gives the effects of alter characteristics on the probability of allocating to alters. The bottom block of estimates gives the effects of dyadic characteristics on the probability of allocating to alters. Each estimate gives the effect of a single predictor controlling for all other predictors of that outcome. These estimates are standardized such that each estimate in a given model has an equal width 90% credible region.

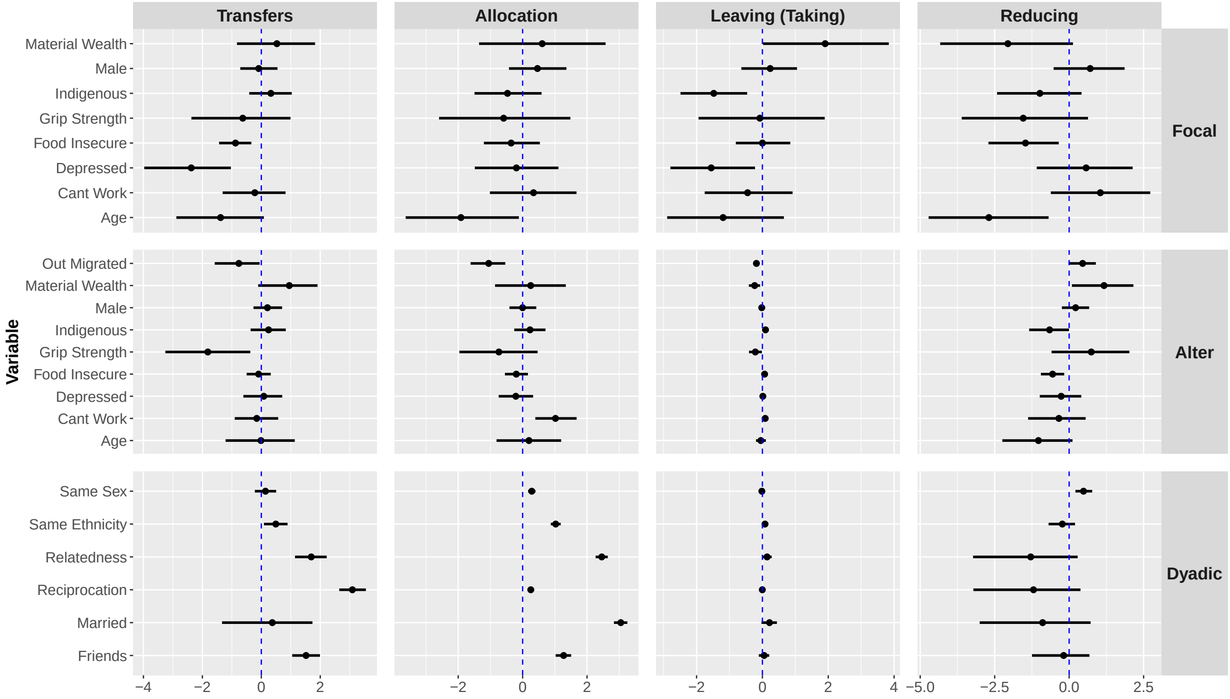

Figure 4: Multinomial regression results (unstandardized coefficients) from the simplified SRM. Multivariate model estimates (medians and 90% credible regions) of predictors of allocations to alters. Each column indicates an outcome variable: from left to right, i) resource transfers over the 30 days prior to the survey, ii) coin allocations in the allocation game, iii) coin allocations in the taking game (coded so that positive parameter estimates reflect *leaving* coins), and iv) token allocations in the reducing game. The top block of estimates in each model gives the effects of focal characteristics on the probability of allocating to alters. The second block of estimates gives the effects of alter characteristics on the probability of allocating to alters. The bottom block of estimates gives the effects of dyadic characteristics on the probability of allocating to alters. Each estimate gives the effect of a single predictor controlling for all other predictors of that outcome.

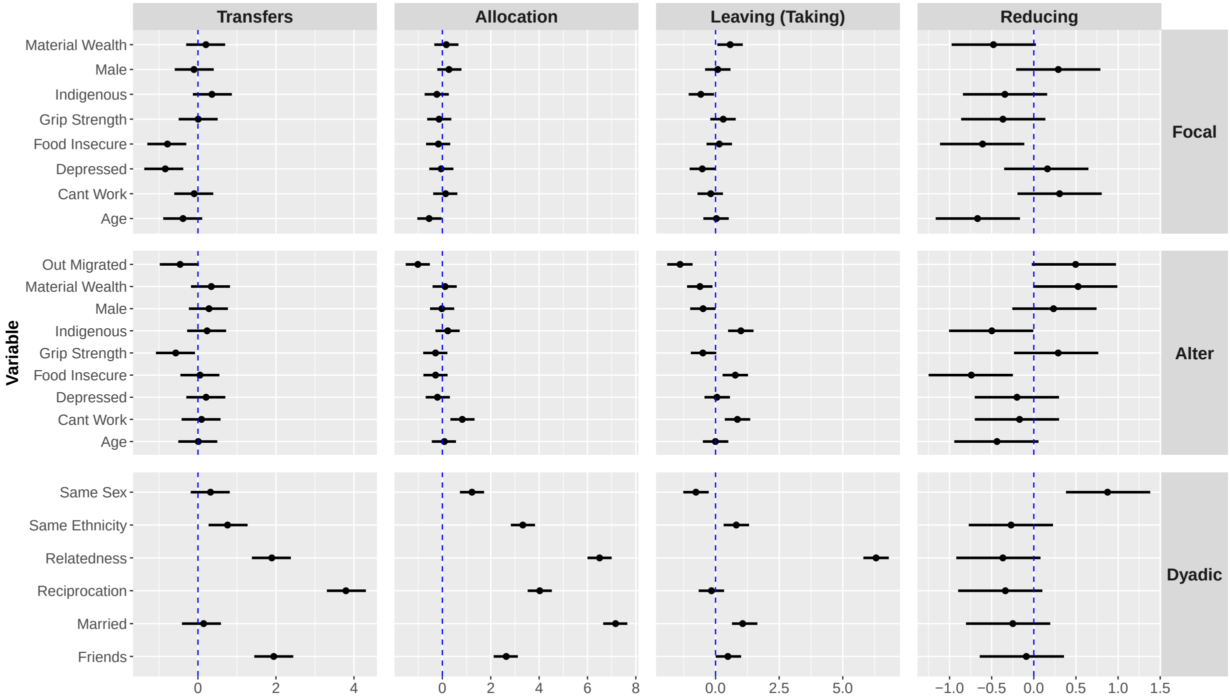

Figure 5: Truncated multinomial regression results (standardized coefficients) from the simplified SRM. Multivariate model estimates (medians and 90% credible regions) of predictors of allocations to alters. Each column indicates an outcome variable: from left to right, i) resource transfers over the 30 days prior to the survey, ii) coin allocations in the allocation game, iii) coin allocations in the taking game (coded so that positive parameter estimates reflect *leaving* coins), and iv) token allocations in the reducing game. The top block of estimates in each model gives the effects of focal characteristics on the probability of allocating to alters. The second block of estimates gives the effects of alter characteristics on the probability of allocating to alters. The bottom block of estimates gives the effects of dyadic characteristics on the probability of allocating to alters. Each estimate gives the effect of a single predictor controlling for all other predictors of that outcome. These estimates are standardized such that each estimate in a given model has an equal width 90% credible region.

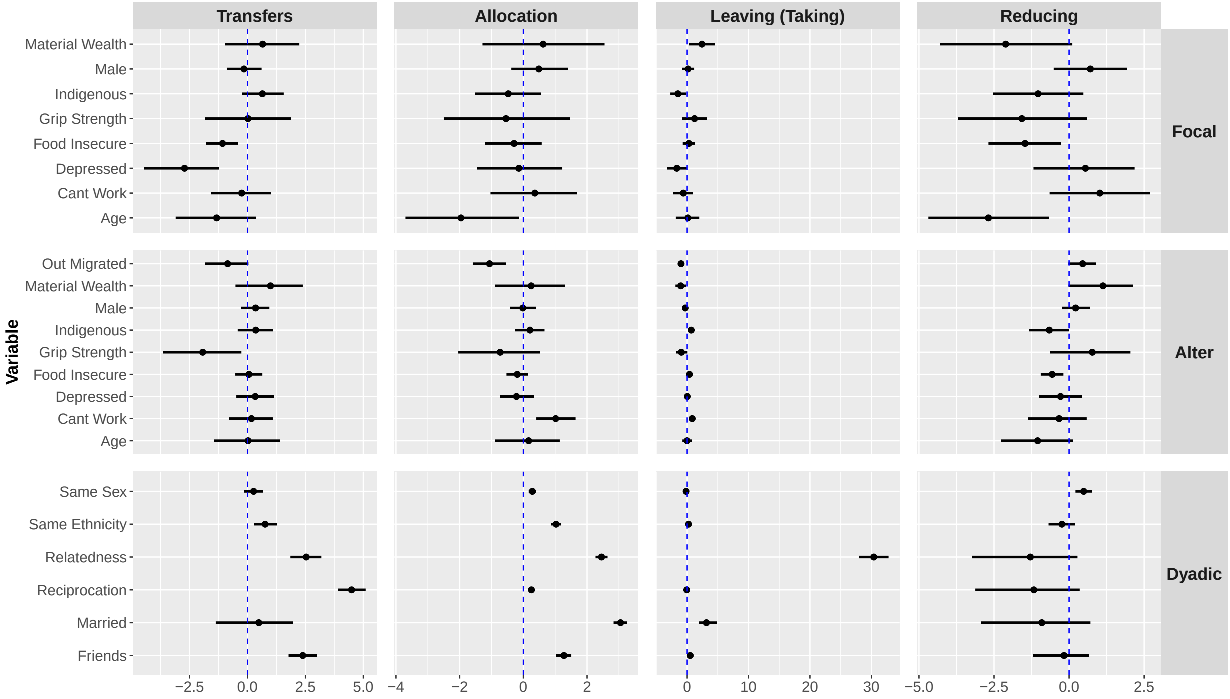

Figure 6: Truncated multinomial regression results (unstandardized coefficients) from the simplified SRM. Multivariate model estimates (medians and 90% credible regions) of predictors of allocations to alters. Each column indicates an outcome variable: from left to right, i) resource transfers over the 30 days prior to the survey, ii) coin allocations in the allocation game, iii) coin allocations in the taking game (coded so that positive parameter estimates reflect *leaving* coins), and iv) token allocations in the reducing game. The top block of estimates in each model gives the effects of focal characteristics on the probability of allocating to alters. The second block of estimates gives the effects of alter characteristics on the probability of allocating to alters. The bottom block of estimates gives the effects of dyadic characteristics on the probability of allocating to alters. Each estimate gives the effect of a single predictor controlling for all other predictors of that outcome.

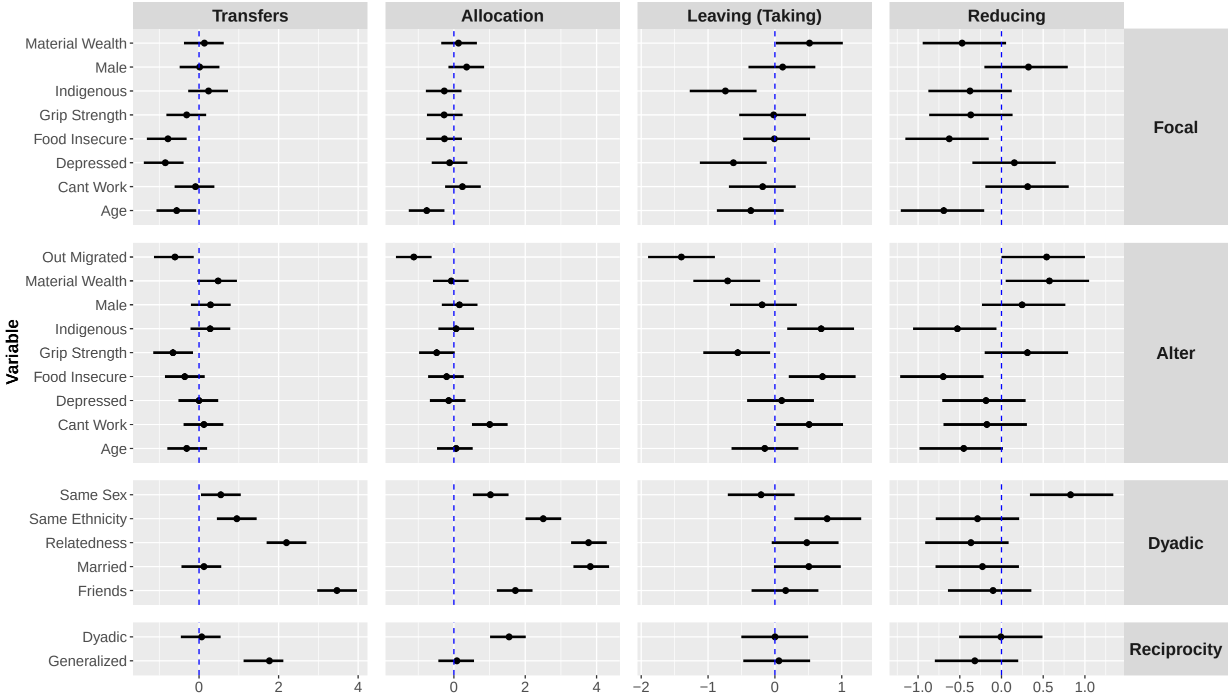

Figure 7: Standard multinomial regression results (standardized coefficients) from the full SRM. Multivariate model estimates (medians and 90% credible regions) of predictors of allocations to alters. Each column indicates an outcome variable: from left to right, i) resource transfers over the 30 days prior to the survey, ii) coin allocations in the allocation game, iii) coin allocations in the taking game (coded so that positive parameter estimates reflect *leaving* coins), and iv) token allocations in the reducing game. The top block of estimates in each model gives the effects of focal characteristics on the probability of allocating to alters. The second block of estimates gives the effects of alter characteristics on the probability of allocating to alters. The bottom block of estimates gives the effects of dyadic characteristics on the probability of allocating to alters. Each estimate gives the effect of a single predictor controlling for all other predictors of that outcome. These estimates are standardized such that each estimate in a given model has an equal width 90% credible region.

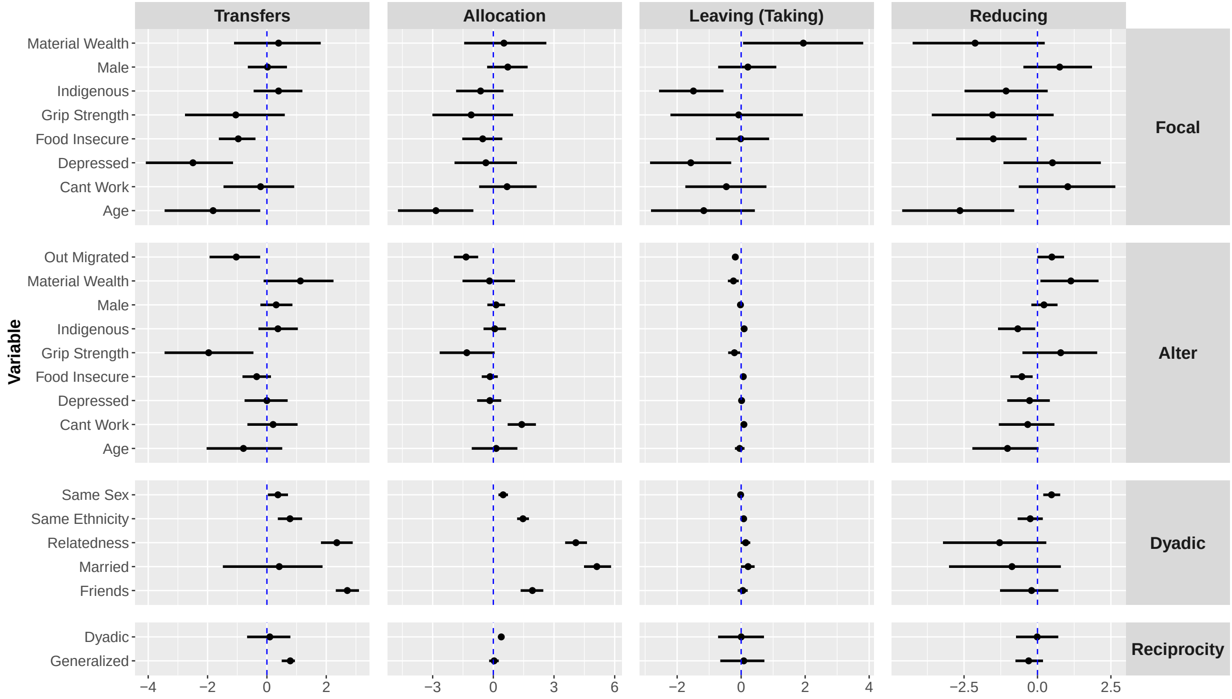

Figure 8: Standard multinomial regression results (unstandardized coefficients) from the full SRM. Multivariate model estimates (medians and 90% credible regions) of predictors of allocations to alters. Each column indicates an outcome variable: from left to right, i) resource transfers over the 30 days prior to the survey, ii) coin allocations in the allocation game, iii) coin allocations in the taking game (coded so that positive parameter estimates reflect *leaving* coins), and iv) token allocations in the reducing game. The top block of estimates in each model gives the effects of focal characteristics on the probability of allocating to alters. The second block of estimates gives the effects of alter characteristics on the probability of allocating to alters. The bottom block of estimates gives the effects of dyadic characteristics on the probability of allocating to alters. Each estimate gives the effect of a single predictor controlling for all other predictors of that outcome.

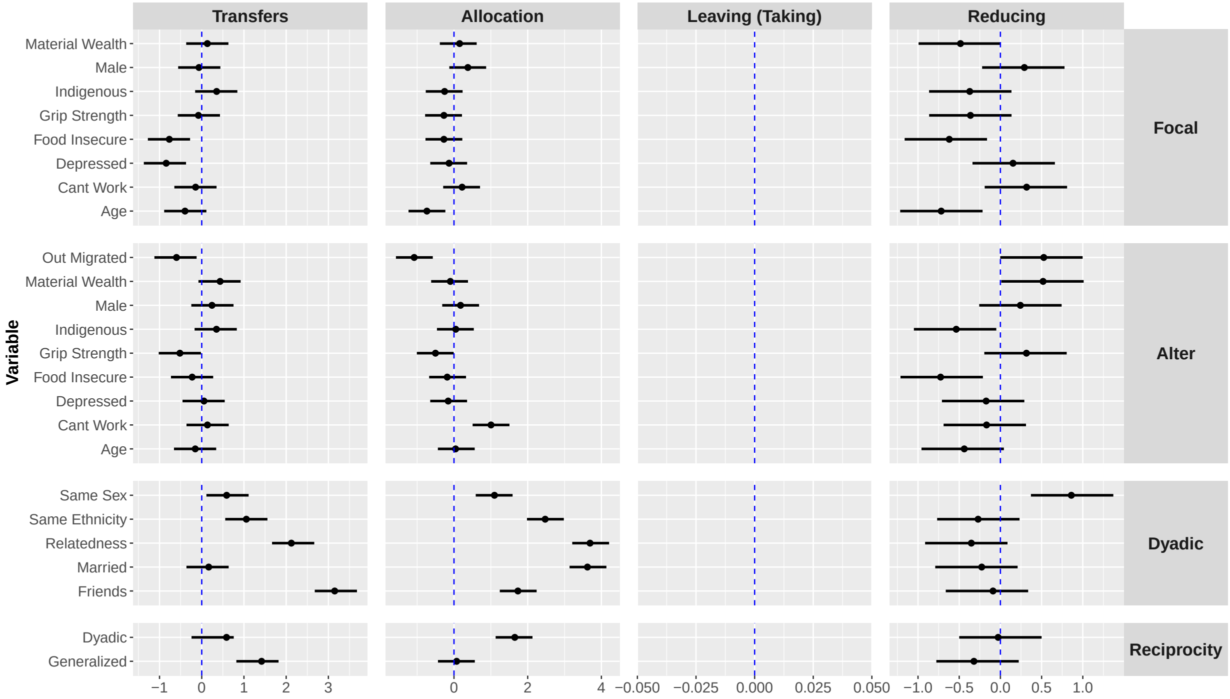

Figure 9: Truncated multinomial regression results (standardized coefficients) from the full SRM. Multivariate model estimates (medians and 90% credible regions) of predictors of allocations to alters. Each column indicates an outcome variable: from left to right, i) resource transfers over the 30 days prior to the survey, ii) coin allocations in the allocation game, iii) coin allocations in the taking game (coded so that positive parameter estimates reflect *leaving* coins), and iv) token allocations in the reducing game. The top block of estimates in each model gives the effects of focal characteristics on the probability of allocating to alters. The second block of estimates gives the effects of alter characteristics on the probability of allocating to alters. The bottom block of estimates gives the effects of dyadic characteristics on the probability of allocating to alters. Each estimate gives the effect of a single predictor controlling for all other predictors of that outcome. These estimates are standardized such that each estimate in a given model has an equal width 90% credible region. Note that the taking/leaving model could not be reliably estimated using the full SRM and the truncated multinomial outcome distribution, due to a difficult posterior geometry that led to many divergent MCMC transitions.

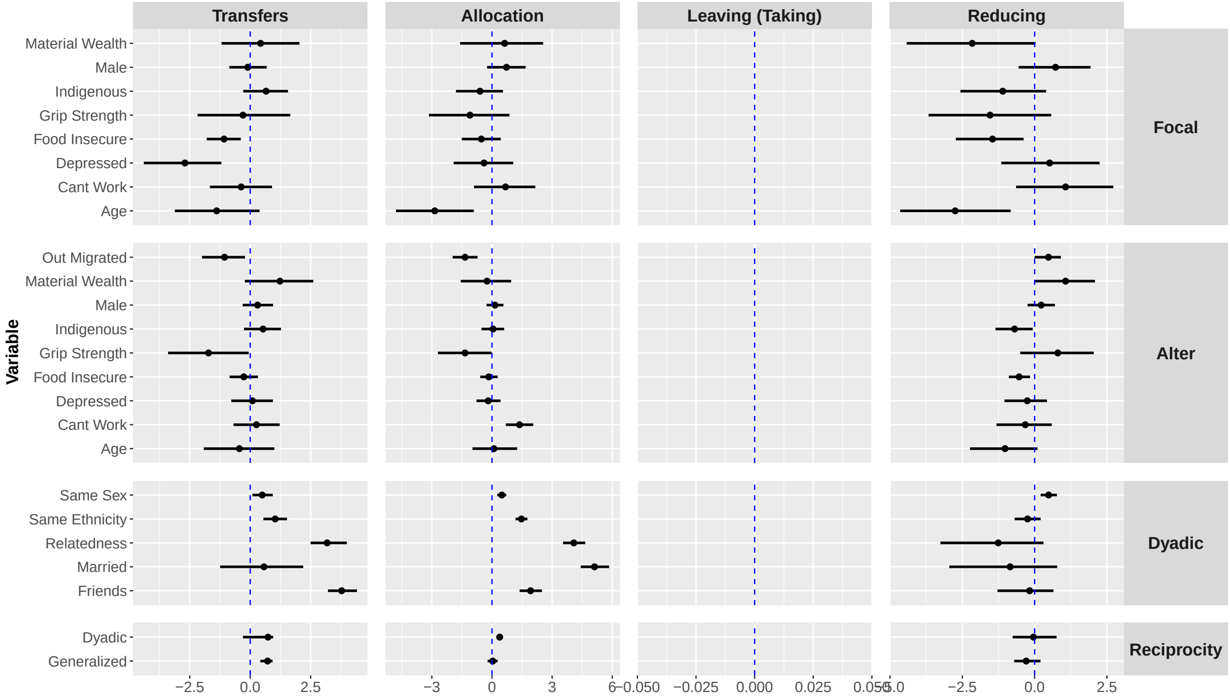

Figure 10: Truncated multinomial regression results (unstandardized coefficients) from the full SRM. Multivariate model estimates (medians and 90% credible regions) of predictors of allocations to alters. Each column indicates an outcome variable: from left to right, i) resource transfers over the 30 days prior to the survey, ii) coin allocations in the allocation game, iii) coin allocations in the taking game (coded so that positive parameter estimates reflect *leaving* coins), and iv) token allocations in the reducing game. The top block of estimates in each model gives the effects of focal characteristics on the probability of allocating to alters. The second block of estimates gives the effects of alter characteristics on the probability of allocating to alters. The bottom block of estimates gives the effects of dyadic characteristics on the probability of allocating to alters. Each estimate gives the effect of a single predictor controlling for all other predictors of that outcome. Note that the taking/leaving model could not be reliably estimated using the full SRM and the truncated multinomial outcome distribution, due to a difficult posterior geometry that led to many divergent MCMC transitions.

as between friends or kin. There is also a strong signal of generalized reciprocation (individuals with high rates of giving to others were also targets of giving, holding constant other factors, but not necessarily by the same community members that they themselves gave to). Note that the absence of a positive effect of marriage on a transfer is due to the transfer question being framed specifically in terms of inter-household transfers.

Allocation game In the second column, top block, we see that the elderly were less likely to give coins to others. In the next block, we see that those individuals who cannot work were more likely to be allocated coins. In contrast, those who out-migrated were less likely to be allocated coins. Finally, we see in the third block that a coin allocation is more likely between individuals of the same sex or ethnicity, as well as between friends, spouses, or kin. There is also a strong signal of dyadic reciprocation (holding other factors constant, individuals are more likely to give coins to alters who also gave to them).

Taking game In the third column, top block, we see that the materially wealthy were more likely to leave coins for others in the taking game. In contrast, indigenous individuals and those who reported symptoms of depression were less likely to leave coins for others. In the next block, we see that indigenous individuals, the food insecure, and those who cannot work were more likely to be left coins. In contrast, those who out-migrated, the materially wealthy, and those with high grip strength were less likely to be left with coins. Finally, we see in the third block that a coin is more likely to be left for an alter if the pair is of the same ethnicity, related, or married.

Costly reduction game Finally, in the fourth column, top block, we see that the elderly and food insecure were less likely to pay to punish others. In the next block, we see that the materially wealthy and those who out-migrated were more likely to be punished. In contrast, indigenous individuals, the elderly, and food insecure individuals were less likely to be punished by others. Finally, we see in the third block that punishment is more likely to occur between same-sex dyads.

5 Discussion

The results of this analysis—both those based on self-reported giving and those based on experimental RICH games—are in line with a range of anthropological studies demonstrating that kinship[3,8–10], reciprocity[11–17], and need-based heuristics[18–21] are predictive of cooperation and costly resource transfers.

Importantly for our purposes, the use of both self-reported resources transfers and network structured economic games among the same sample of individuals allows us to compare and contrast the methods, and comment on the utility of each in explaining human behavior.

RICH economic games can be a useful tool for anthropologists, economists, and psychologists, as such games have comparably high external validity, relative to other economic games. Note, for example, the parallels between the predictors of self-reported transfers and behavior in the RICH games; dyadic predictors like kinship, friendship, coethnicity, and, to some extent, reciprocation are consistent across outcome measures.

On the other hand, there are many more statistically reliable predictors of experimental allocations than self-reported resource transfers. This potentially indicates that respondents in the economic games are acting on preferences that they are not able to express in daily life,

due to at least one of a variety of constraints (e.g., financial resource availability, which can be experimentally relaxed by allocating coins to respondents). If we were to rely strictly on analysis of empirical resource transfers, we could miss many of the preferences underlying inter-personal relationship formation and maintenance.

Rather than attending to the highly constrained products of individuals interacting in social systems, economic games are often designed to measure the comparatively less constrained preferences of individuals. As such, experiments like these are not replacements for observational studies of behavior. However, by measuring both the socio-ecologically constrained realizations of preferences (self-report or observational studies) and less constrained direct measures of preferences (game behavior), researchers can learn more about how socio-cultural institutions shape social dynamics and thus better appreciate the contours of social life.

1. Gervais, M. M. RICH economic games for networked relationships and communities: Development and preliminary validation in Yasawa, Fiji. *Field Methods* **29**, 113–129 (2017).

2. Furukawa, T. A., Kessler, R. C., Slade, T. & Andrews, G. The performance of the K6 and K10 screening scales for psychological distress in the Australian National Survey of Mental Health and Well-Being. *Psychological Medicine* **33**, 357–362 (2003).

3. Koster, J. M. & Leckie, G. Food sharing networks in lowland Nicaragua: an application of the social relations model to count data. *Social Networks* **38**, 100–110 (2014).

4. McElreath, R. *Statistical rethinking: A Bayesian course with examples in R and Stan* (CRC press, 2020).

5. R Core Team. *R: A Language and Environment for Statistical Computing*. R Foundation for Statistical Computing, Vienna, Austria (2019). URL `https://www.R-project.org/`.

6. Stan Development Team. Stan: A C++ Library for Probability and Sampling, Version 2.18.2 (2015). URL `http://mc-stan.org/`.

7. Gelman, A. *et al.* *Bayesian data analysis* (CRC Press, 2014).

8. Gurven, M., Allen-Arave, W., Hill, K. & Hurtado, A. M. Reservation food sharing among the Ache of Paraguay. *Human Nature* **12**, 273–297 (2001).

9. Hooper, P. L., Gurven, M., Winking, J. & Kaplan, H. S. Inclusive fitness and differential productivity across the life course determine intergenerational transfers in a small-scale human society. *Proceedings of the Royal Society of London B: Biological Sciences* **282**, 20142808 (2015).

10. Thomas, M. G. *et al.* Kinship underlies costly cooperation in Mosuo villages. *Royal Society Open Science* **5**, 171535 (2018).

11. Gurven, M. Reciprocal altruism and food sharing decisions among Hiwi and Ache hunter–gatherers. *Behavioral Ecology and Sociobiology* **56**, 366–380 (2004).

12. Nolin, D. A. Food-sharing networks in Lamalera, Indonesia. *Human Nature* **21**, 243–268 (2010).

13. Jaeggi, A. V., Hooper, P. L., Beheim, B. A., Kaplan, H. & Gurven, M. Reciprocal exchange patterned by market forces helps ex-

plain cooperation in a small-scale society. *Current Biology* **26**, 2180–2187 (2016).

14. Ziker, J. P., Rasmussen, J. & Nolin, D. A. Indigenous Siberians solve collective action problems through sharing and traditional knowledge. *Sustainability Science* **11**, 45–55 (2016).

15. Ready, E. & Power, E. A. Why wage earners hunt: Food sharing, social structure, and influence in an Arctic mixed economy. *Current Anthropology* **59**, 74–97 (2018).

16. Koster, J., Leckie, G., Miller, A. & Hames, R. Multilevel modeling analysis of dyadic network data with an application to Ye'kwana food sharing. *American Journal of Physical Anthropology* **157**, 507–512 (2015).

17. Power, E. A. Social support networks and religiosity in rural South India. *Nature Human Behaviour* **1**, 0057 (2017).

18. Koster, J. Interhousehold meat sharing among Mayangna and Miskito horticulturalists in Nicaragua. *Human Nature* **22**, 394–415 (2011).

19. Caudell, M., Rotolo, T. & Grima, M. Informal lending networks in rural Ethiopia. *Social Networks* **40**, 34–42 (2015).

20. Aktipis, A. *et al.* Cooperation in an uncertain world: for the Maasai of East Africa, need-based transfers outperform account-keeping in volatile environments. *Human Ecology* **44**, 353–364 (2016).

21. Hao, Y., Armbruster, D., Cronk, L. & Aktipis, C. A. Need-based transfers on a network: a model of risk-pooling in ecologically volatile environments. *Evolution and Human Behavior* **36**, 265–273 (2015).
